# Otolith‐based age determination in *Anguilla anguilla* (Linnaeus 1758) from Lake Vistonida, NE Greece: A modified Crack and Burn protocol

**DOI:** 10.1111/jfb.70555

**Published:** 2026-07-07

**Authors:** Paraskevi Papadopoulou, Argyriοs Sapounidis, Manos Koutrakis, Ioannis D. Leonardos

**Affiliations:** ^1^ Hellenic Agricultural Organization‐DIMITRA Fisheries Research Institute Kavala Greece; ^2^ Laboratory of Zoology, Biological Applications and Technology Department University of Ioannina Ioannina Greece

**Keywords:** age estimation, FRI‐modified Crack and Burn protocol, otolith

## Abstract

This study presents a modified otolith preparation protocol to estimate the age of *Anguilla* from Lake Vistonida (NE Greece). In experimental attempts, 300 otoliths were processed using standard and modified eel ageing methods, producing low success rates (0%–20%) due to poor annuli visibility and structural damage. To overcome these limitations, a ten‐step Fisheries Research Institute (FRI) modified Crack and Burn protocol, representing a species‐specific adaptation of existing Crack & Burn methodologies, was developed, combining thermal treatment, polishing, acid bath and glycerin application. This method achieved 90% success in age determination and provided a reliable method for age reading (CV = 1.97).

European eel *Anguilla* (Linnaeus, 1758) has one of the widest distributions across Europe, North Africa, the Mediterranean and Black Sea (Tesch, 2003). This broad range allows local eel populations to adapt to specific environmental conditions (temperature, diet, latitude, individual traits, habitat), resulting in diverse growth patterns within the species (Vøllestad, Jonsson, et al., 1988; Vøllestad, Lecomte‐Finiger, et al., 1988). Over the years, numerous studies have focused on understanding the unique life cycle of the eel (Aida et al., 2003), including studies of their biology (length‐weight relations, age determination etc.), ecology and reproductive migration (Aarestrup et al., 2009).

Age estimation of the eel has been extensively studied (Andrews et al., 2025; ICES 2009a, 2009b, 2011), leading to the development of various methodological approaches following the initial proposal to use otoliths to estimate the age of eels (Andrews et al., 2025; Campana, 2001; ICES 2009a, 2009b, 2011; Melià et al., 2006; Moriarty, 1983). The ICES Workshop on Age Reading of European and American Eel (ICES, 2009a, 2009b, 2011) suggested that the most effective methods for processing otoliths of European eels are (i) the grinding and polishing protocol, (ii) the burning and cracking protocol and (iii) the whole otolith clearing protocol ‘in toto’.

The fundamental principle of age estimation using otoliths is the counting of annual rings, which represent periods of fast and slow growth throughout the eel's lifespan. These bands reflect the eel's growth and are influenced by factors such as temperature, dietary habits, latitude, the characteristics of the individuals, etc. (Andrews et al., 2025; ICES, 2019; Vøllestad, Jonsson, et al., 1988; Vøllestad, Lecomte‐Finiger, et al., 1988). Studies on eel age across Europe indicate that eels in northern regions tend to grow more slowly and live longer (Durif et al., 2020; Poole & Reynolds, 1998), compared to those in southern Europe, particularly in the Mediterranean, where they grow faster and reach maturity at a younger age (Correia et al., 2021; ICES, 2019; Tahri & Panfili, 2023). There are still uncertainties, mainly due to the lack of a common validation method across regions and the challenges of applying the same otolith processing protocol to eel otoliths from different regions across Europe (Durif et al., 2020).

Concerns have been raised regarding the suitability of applying the same otolith processing protocol to eel otoliths from different regions across Europe, as significant variations in eel age estimates have been observed depending on the method used (ICES, 2019; Poole & Reynolds, 1998). For this reason, variations in the steps of each protocol are necessary to achieve the highest resolution of annual rings in the otoliths of the European eel. Given these limitations and the biological particularities of the species, this study aims to evaluate and compare the most used otolith ageing methods and to develop a modified, region‐specific protocol that ensures the production of reliable, repeatable and high‐quality otolith images/processed samples. The proposed protocol can produce otolith images where the core is clearly distinguishable and where true annuli can be reliably differentiated from false rings or summer checks.

To address these limitations, 869 eel otoliths from Lake Vistonida (Northeastern Greece), part of the ‘Otolith Library’ of the Fisheries Research Institute, were analysed in this study. Of these, 300 otoliths were used in experimental trials to determine the most suitable methodological approach for analysis. Specifically, 50 otoliths were processed with each of the following methods: Grinding and Polishing, Crack and Burn, and the Whole Otolith Clearing protocol (‘in toto’), as described in the ICES WKAREA Reports (2009a, 2009b, 2011). Additionally, two modified versions of the Crack and Burn method (Version 1 and Version 2) were each applied to 50 otoliths. The first modification involved replacing the open flame with a heated plate set to a stable temperature of 400°C. The use of a heated plate was first introduced by Power (1978), who applied it to improve the visibility of annuli near the edge of *Salvelinus namaycush* otoliths. Many years later, hot plate heating was reintroduced in the standardized sBCB protocol for age determination of *Capros aper*, where whole otoliths were burned at 370°C (Vagenas et al., 2021). The second modification combined the use of the heated plate with staining the burned otolith surfaces using a plain neutral red solution (3.3 g/L).

Further tests and modifications of the basic ‘Crack and Burn’ protocol and the sBCB protocol (Vagenas et al., 2021) were performed on 50 otoliths, leading to the development of the ‘Fisheries Research Institute (FRI) modified Crack and Burn protocol’. This protocol consists of ten steps: (i) Otolith Drying: The otoliths were initially dried in an oven at 60°C to remove any residual moisture. (ii) Cracking: The dried otoliths were cracked near the core. (iii) Otolith Drying: The cracked otoliths were dried again in an oven at 60°C to remove the residual moisture. (iv) Burning: Both halves of the cracked otolith were burned on a hotplate at 400°C. (v) Cooling: After burning, the otoliths were allowed to cool to room temperature. (vi) Cleaning and Polishing: The broken surfaces were cleaned and polished using 1000 and 2000 grit grinding paper. (vii) Acid Wash: The polished surfaces were washed with 1 drop of 1% v/v hydrochloric acid (HCl) to further enhance the visibility of the annual rings (Vøllestad, Jonsson, et al., 1988; Vøllestad, Lecomte‐Finiger, et al., 1988). (viii) Repetition: Steps 3 to 7 were repeated until the highest resolution of the annual rings was achieved. (ix) Microscopic Observation: The otoliths were observed under a stereoscope with one drop of glycerin. (x) Photographic Documentation: High quality photographs were taken for further analysis. The processing and photography of the otoliths were carried out using the NIKON SMZ18 stereoscopic system, equipped with a DS‐Fi3 model camera and Control Unit L4.

The Otolith Uncertainty Index (OUI) was applied to quantify differences in age readings among the two readers. The OUI classifies discrepancies into three levels: level 1 (<3 years), level 2 (3–5 years) and level 3 (>5 years) (Durif et al., 2020), alongside the Coefficient of Variation (CV; Chang, 1982; Campana, 2001), which is expressed as the ratio of the standard deviation over the mean:

$${\mathrm{CV}}_{j}=100\%x\frac{\sqrt{\sum_{i=1}^{R}\frac{{\left(X_{\mathrm{ij}}-X_{j}\right)}^{2}}{R-1}}}{X_{j}}$$

where *X*_*ij*_ is the *i*th age estimate for the *j*th fish, *X*_*j*_ is the mean estimated age for that fish and *R* is the number of times each fish is aged. Lower CV values indicate higher precision among repeated age estimates for all fish. CV for each reader was calculated using the FSA package in R (Ogle, 2013).

During the processing of otoliths from Lake Vistonida, they were found to be highly fragile, frequently exhibiting cracks and scratches extending through the core. This fragility complicated their preparation, as samples often fractured completely, split into irregularly shaped sections or pieces from the edges were chipped off. Specifically, in the Grinding and Polishing protocol, only three out of 50 otoliths were prepared and used for age estimation (Figure 1a). It was observed that most of the otoliths were highly curved, and during grinding, the rings at the edges were lost or the samples were fractured due to fragility issues. The images of the otoliths used for age estimation were of poor quality, resulting in unclear outcomes.

**FIGURE 1:**
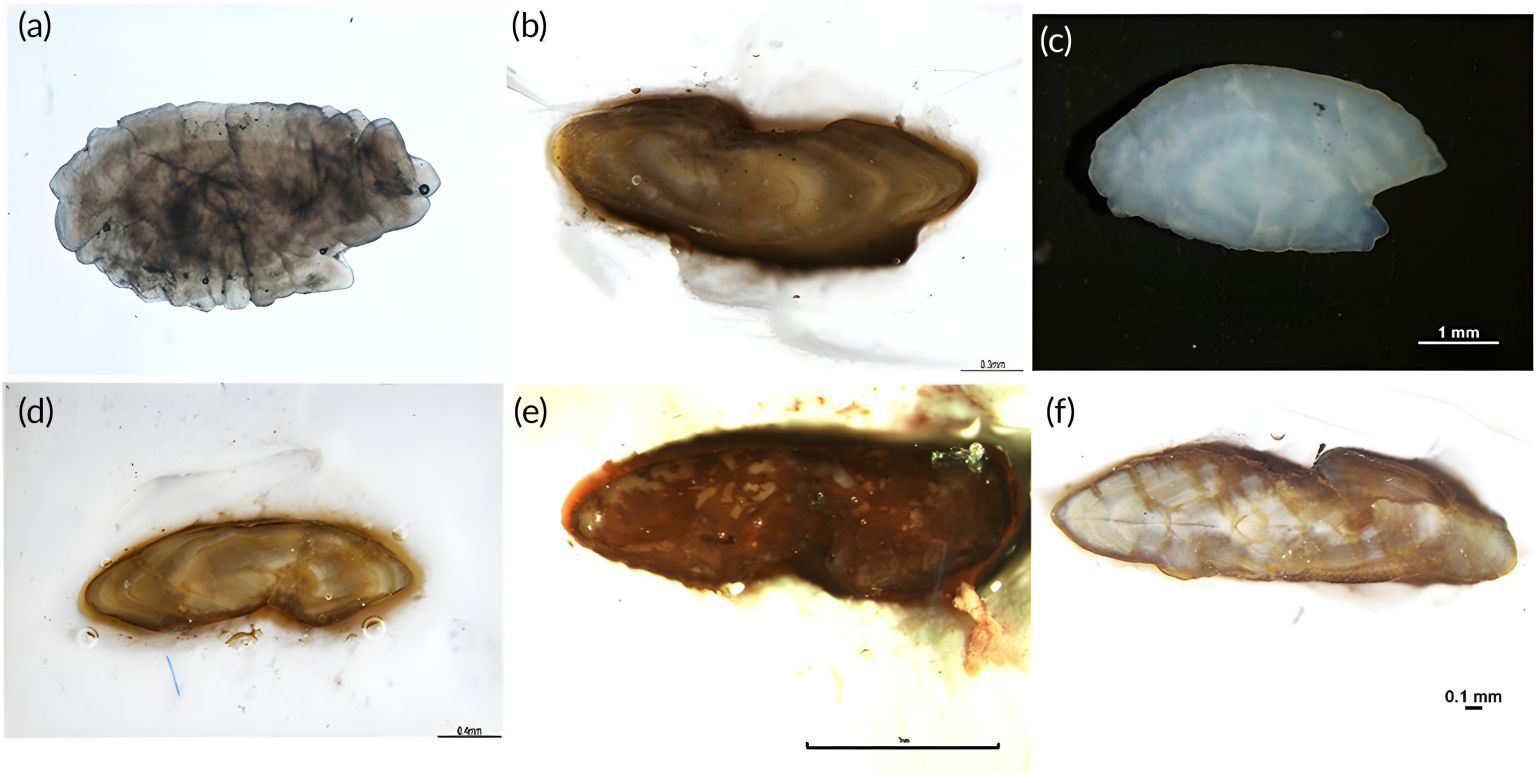
Processed eel otoliths using different protocols during the present study: (a) Grinding and Polishing protocol, (b) Crack and Burn protocol, (c) Whole Otolith Clearing protocol (d) Version 1 modification of Crack and Burn protocol, (e) Version 2 modification of Crack and Burn protocol and (f) FRI modified Crack and Burn protocol.

Crack and Burn protocol yielded five otoliths suitable for age estimation (Figure 1b). Excessive burning was frequently observed in most samples, leading to the loss of annual rings or the disappearance of outer edge annuli. These observations are consistent with the findings of McKeefry et al. (2023), who examined the influence of otolith preparation techniques and reader experience on age estimation accuracy in *Coregonus clupeaformis*. In several cases, it was also not possible to cut through the centre of the otolith, resulting in poor‐quality outcomes.

None of the otoliths processed using the Whole Otolith Clearing protocol provided high‐quality images for age estimation (Figure 1c), the growth rings were not clearly visible across the entire otolith due to the ‘scratches’ already existing on their surface and the thickness of the otoliths themselves, resulting in ambiguous outcomes.

Regarding the modifications of the Crack and Burn protocol, Version 1 (Figure 1d) produced ten otoliths suitable for age estimation, as most of the samples were cracked into irregular pieces. Replacing the alcohol burner with a heated plate reduced the over‐burning cases, as the process became more gradual and could be manually controlled by the operator. This modification produced clearer images compared to the classical method. However, the annual rings remained insufficiently distinct, potentially increasing the risk of overestimating the age structure of the population. Additionally, Version 2‐modified protocol (Figure 1e) produced only five otoliths suitable for age estimation, as most of the samples were cracked into irregular pieces and the cut surface exhibited extensive burning associated with the use of neutral red stain (3.3 g/L), regardless of the staining duration. Therefore, these issues limited the method's effectiveness.

FRI‐modified Crack and Burn protocol produced 45 of 50 otoliths suitable for age estimation (Figure 1f). The combination of additional preparation steps produced the best outcomes among the methods tested, in terms of clarity of the otolith core, reliable discrimination between true rings and false rings or summer checks. These improvements were particularly important for Mediterranean eel otoliths, where strong summer growth marks can easily lead to age overestimation among readers (ICES, 2020). Specifically, oven treatment removed any remaining internal moisture of the otolith, facilitating a more controlled burning process. Polishing the cut surface resulted in a smoother finish and improved visibility of both the core and the growth rings. The acid bath was used to remove residual material left from polishing, while the application of a small amount of glycerin enhanced the optical clarity of the otoliths, making the annual rings easier to visualize and distinguish. For this reason, this method is considered successful.

All otoliths prepared using the protocols Grinding and Polishing and Version 2 modification of Crack and Burn protocol were assigned a level 2 OUI Index, reflecting greater variability in age estimations. The otoliths prepared using the Crack and Burn protocol and Version 1 modification of Crack and Burn protocol, OUI Index values both level 1 and 2 were recorded, with most of the samples classified as level 2. This indicates considerable differences between the readers' observation and consequently, uncertainty in age estimation. In contrast, the FRI modified Crack and Burn protocol produced the highest agreement among readers and the lowest uncertainty in age estimation, as the otoliths were assigned a level 1 OUI Index.

Only the FRI modified Crack and Burn protocol achieved a coefficient of variation <5% (CV = 1.97; Table 1), indicating excellent precision and a high degree of agreement in age estimation between readers (Campana, 2001). The high CV values observed in the remaining methods are likely to be due to the limited sample size and production of unreliable and poor‐quality otolith images/processed samples (Figure 1a–e).

**TABLE 1:** Comparison of six otolith preparation protocols (*n* = 50 per method) in terms of the number of otoliths suitable for age estimation and inter‐reader precision (CV, %).

	Grinding and polishing protocol	Crack and Burn protocol	Whole otolith clearing protocol	Version 1 modification of Crack and Burn protocol	Version 2 modification of Crack and Burn protocol	FRI modified Crack and Burn protocol
Number of otoliths for age estimation	3	5	0	10	5	45
CV between readers	28.16	33.33	NA	25.2	26.23	1.97

Abbreviation: NA, not applicable.

As most of these successful outcomes were obtained using the FRI‐modified Crack and Burn protocol (clear images and CV = 1.97), the remaining 589 otoliths were processed using this method, achieving a success rate of 94.91%. Otoliths that could not be used were either broken in a manner that prevented accurate interpretation or exhibited indistinct annual rings. Differences in age reading between the two independent readers ranged from 1 to 2 years, corresponding to OUI Level 1. In these cases, the otoliths were re‐evaluated to ensure the reliability and accuracy of the final age determinations. These otoliths will be used for the determination of the age structure of the Northeastern Greece European eel population.

In conclusion, this study highlights the significant of applying a region‐specific otolith processing methods and recommends the FRI modified Crack and Burn protocol as the most effective approach for age estimation of the European eel from Lake Vistonida, Greece. Thus, this method could be evaluated and implemented to other European eel populations throughout Europe, especially in southern European countries where eels demonstrate faster growth and attain reproductive maturity at an early age.

## AUTHOR CONTRIBUTIONS

Conceptualization: P.P.; methodology: P.P.; data curation: P.P., A.S.; writing—original draft preparation: P.P.; writing—review and editing: P.P., A.S., I.D.L., M.K.; supervision: I.D.L., M.K., A.S. All authors have read and agreed to the published version of the manuscript.

## Data Availability

Data sharing not applicable to this article as no datasets were generated or analysed during the current study.
